# Supplementary material for: Sphingolipids mediate polar sorting of PIN2 through phosphoinositide consumption at the trans-Golgi network
Source: Nat Commun. 2021 Jul 13;12:4267. doi: 10.1038/s41467-021-24548-0 (PMC8277843; doi:10.1038/s41467-021-24548-0)
Supplement: Supplementary file 1 — Supplementary Information [file 41467_2021_24548_MOESM1_ESM.pdf]

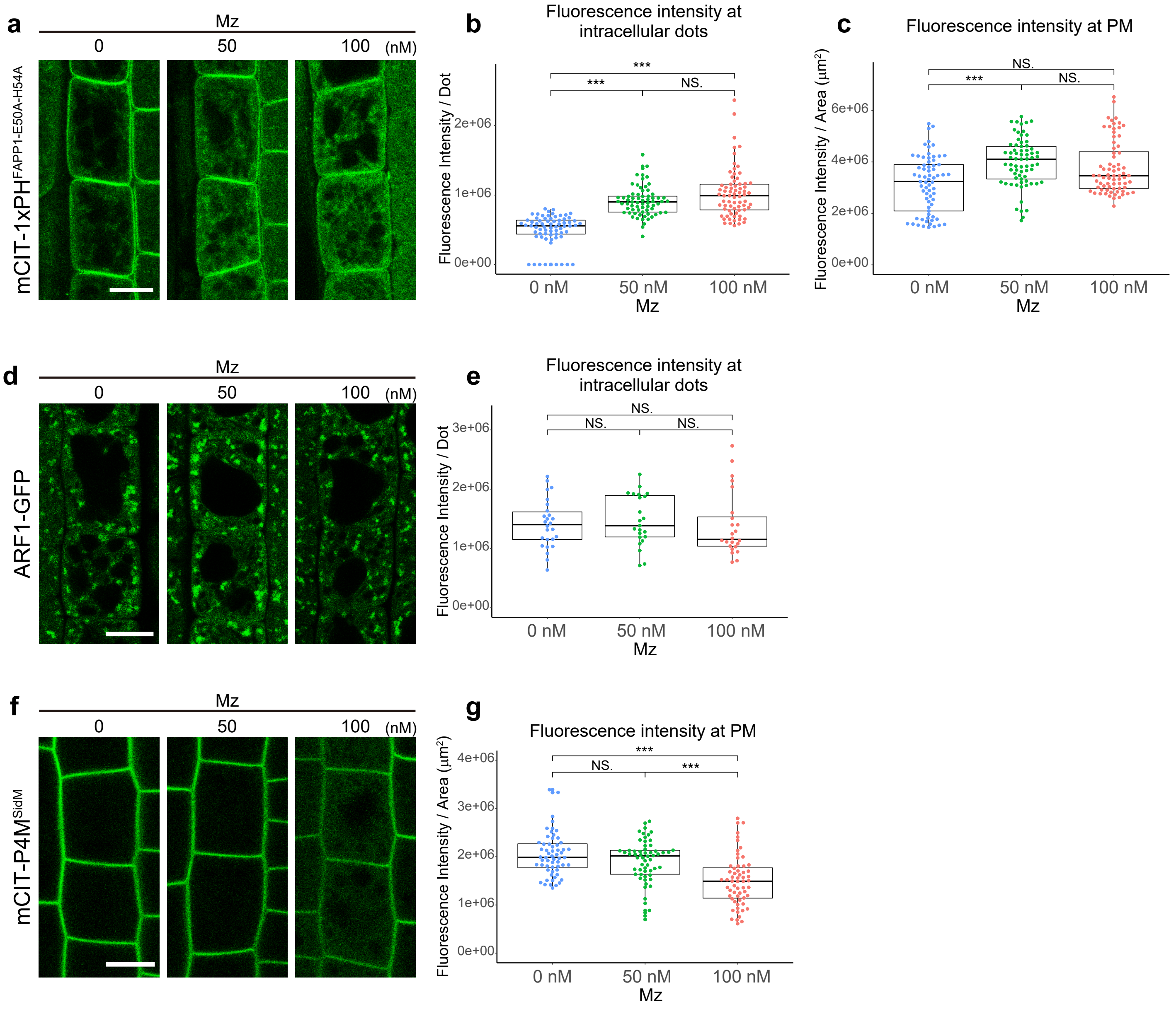

Supplementary Fig. 1. The acyl-chain length of SL is required for the intracellular distribution of PI4P independently of ARF1. (a) Confocal micrographs of root epidermal cells expressing the PI4P sensor mCIT-1xPH$^{FAPP1\text{-}E50A\text{-}E54A}$ upon metazachlor (Mz). Fluorescence intensity at intracellular dots (b) and at the PM (c) was quantified [n = 69 cells from 23 roots (0 and 100 nM) and 72 cells from 24 roots (50 nM)]. (d) Confocal micrographs of root epidermal cells expressing GFP-tagged ARF1. Fluorescence intensity at intracellular dots was quantified [e, n = 26 roots (0 nM), 24 roots (50 nM), 23 roots (100 nM)]. (f) Confocal micrographs of root epidermal cells expressing the PI4P sensor mCIT-P4M^{SidM} upon Mz. The fluorescence intensity at the PM was quantified (g, n = 60 cells from 20 roots). Statistics were done by two-sided Dwass-Steel-Critchlow-Flinger multiple comparison test with Monte Carlo method (10000 iterations), *** *P*-value <0.0001. Each element of the boxplot indicates the following value: center line, median; box limits, the first and third quartiles; whiskers, 1.5x interquartile range; points above or below the whiskers, outliers. Scale bars, 10 μm.

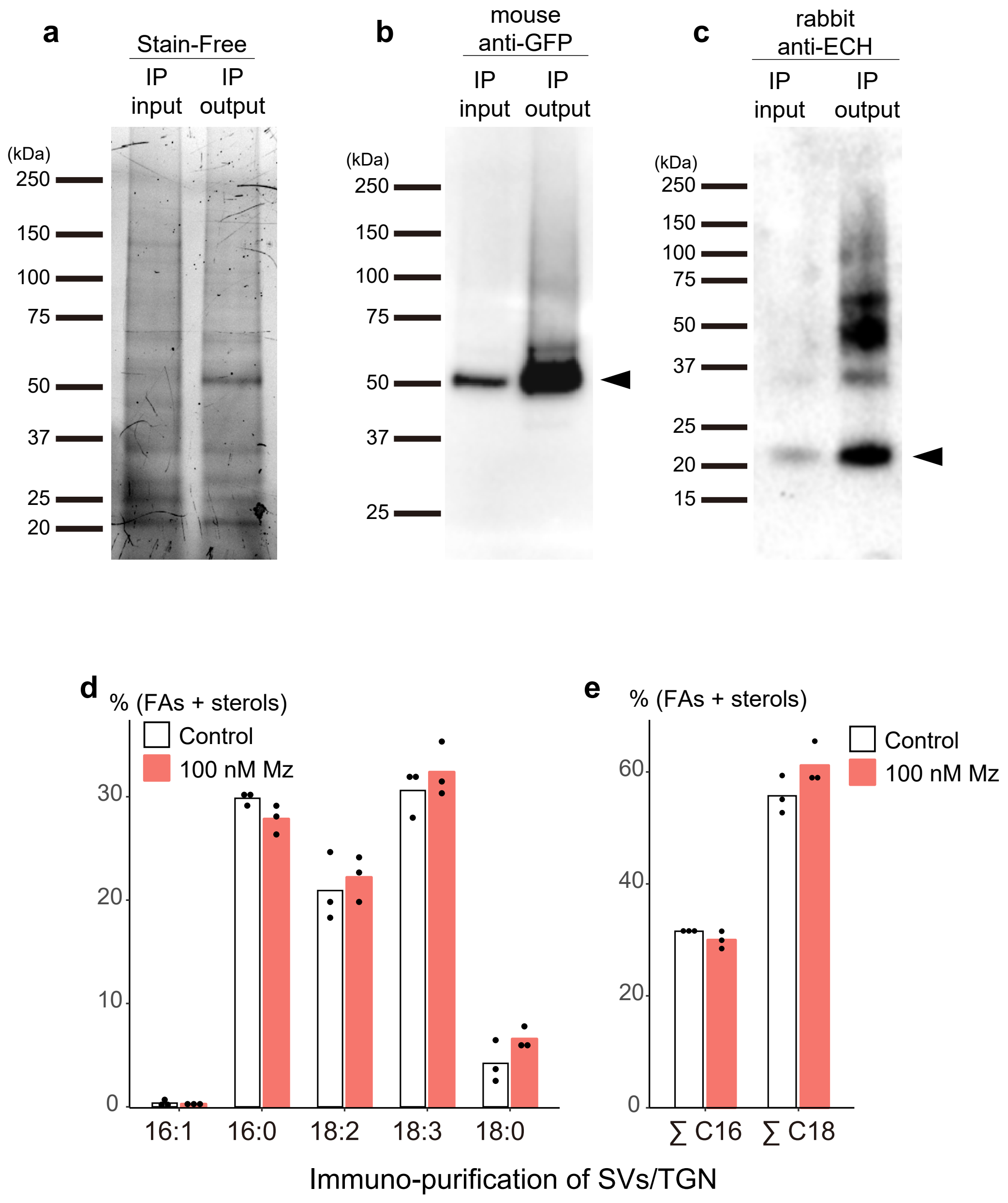

Supplementary Fig. 2. Additional information of figure 3d, e. This is an additional information on lipid analyses of immuno-purified SVs/TGN fraction with or without Mz treatment. (a-c) Immuno-purification of SYP61 SVs/TGN compartments used for lipid analyses and label-free proteomics. (a) Loading control for (b) and (c). IP input and output were loaded on Stain-Free polyacrylamide gel to confirm their equal loading. (b, c) Representative western-blotting images with anti-GFP (b) or anti-ECHIDNA (c) antibodies showing enrichment of SVs/TGN compartments in IP output over the microsomal IP input. Western-blotting were repeated four times with similar results. (d, e) GC-MS analyses of fatty acids in SYP61 SVs/TGN compartments. The pool of C16 and C18, mainly present in phospholipids, is not altered by metazachlor (Mz) as shown in details (d) or by the sum of C16 or the sum of C18 (e). n = 3 biological replicates, the dots show the dispersion of data.

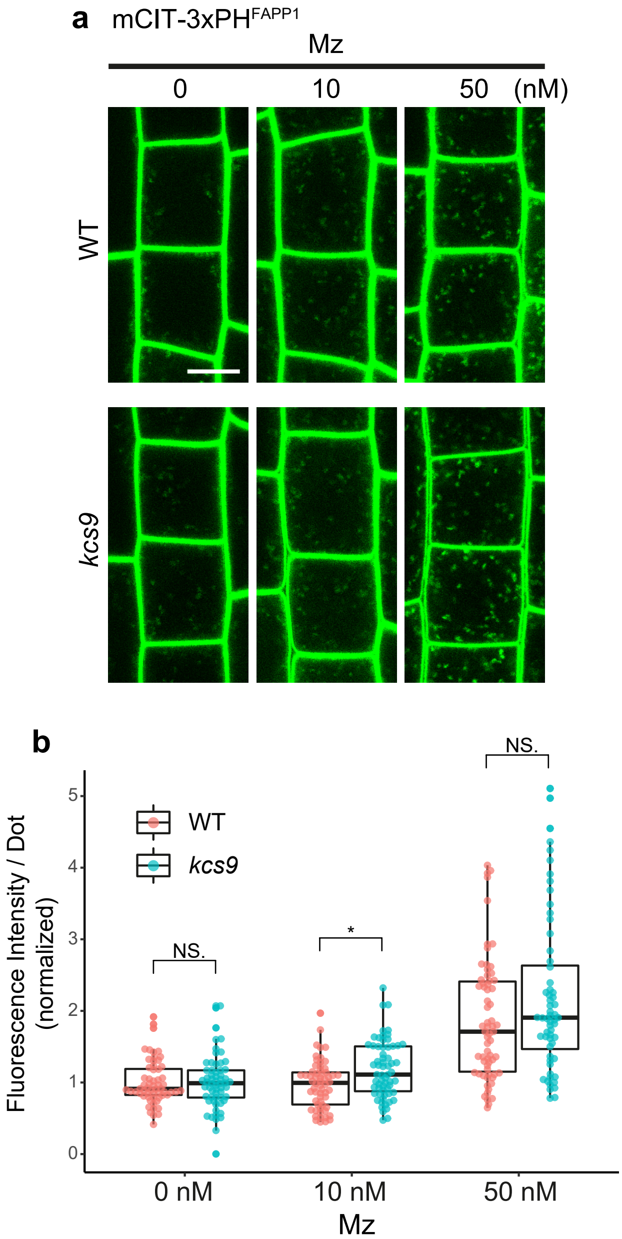

Supplementary Fig. 3. The VLCFA-producing enzyme KCS9 is involved in PI4P accumulation at TGN. (a) Confocal micrographs of wild-type or *kcs9* mutant root epidermal cells expressing the PI4P sensor mCIT-3xPH^{FAPP1} in control condition (0) or treated with 10 nM metazachlor (Mz) or 50 nM Mz. (b) Quantification of the fluorescence intensity at intracellular dots. While in control condition the fluorescence intensity was not significantly different between wild-type and *kcs9* mutant, it became significantly increased in *kcs9* mutant upon 10 nM Mz treatment. n = 60 cells from 20 roots (0 and 10 nM of WT and 0 and 50 nM of *kcs9*), 66 cells from 22 roots (100 nM of WT), 63 cells from 21 roots (10 nM of *kcs9*). Statistics were done by two-sided Wilcoxson's rank-sum test. *P-value<0.01. Each element of the boxplot indicates the following value: center line, median; box limits, the first and third quartiles; whiskers, 1.5x interquartile range; points above or below the whiskers, outliers. Scale bar, 10 µm.

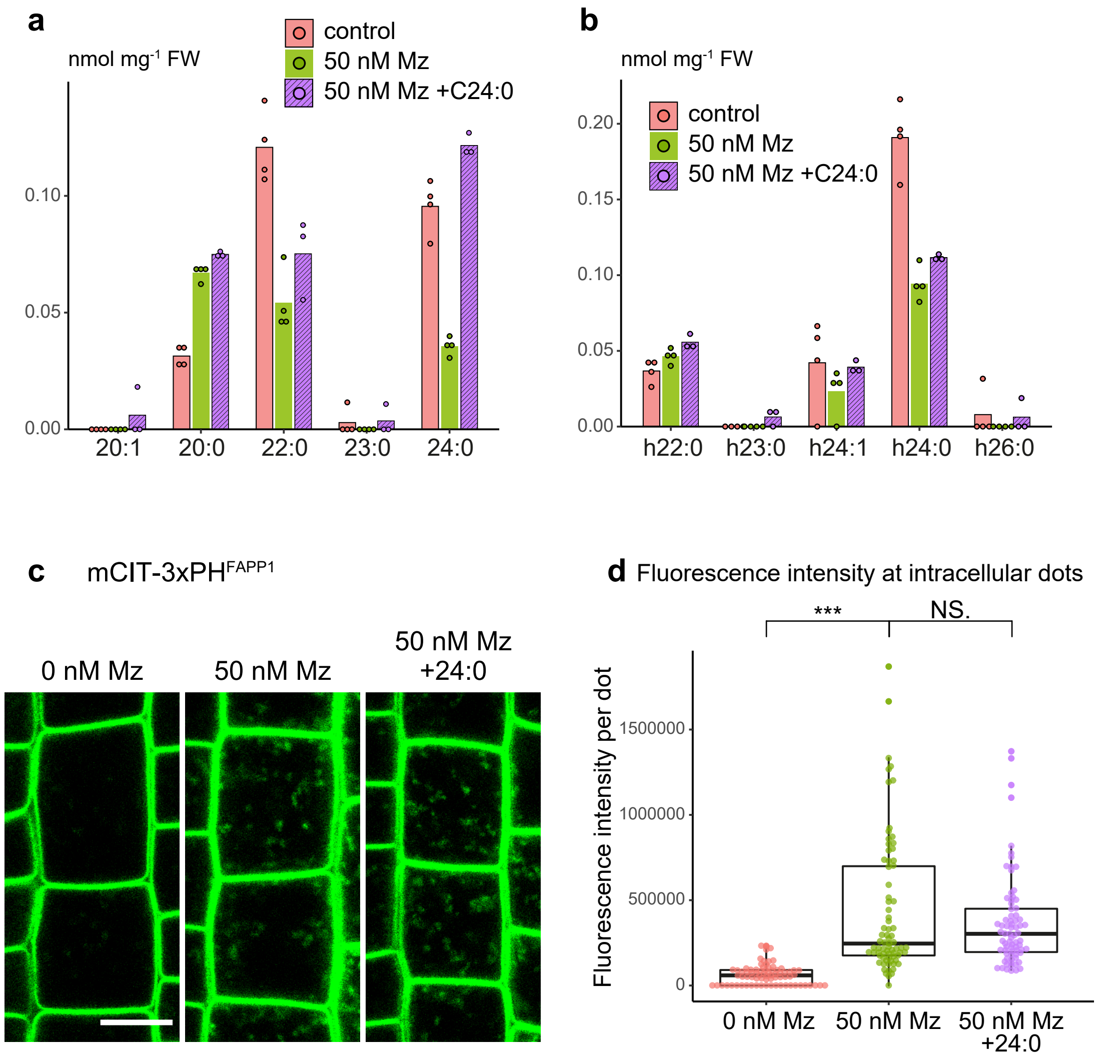

Supplementary Fig. 4. Non-α-hydroxylated 24:0 does not rescue the metazachlor-induced PI4P distribution defect at TGN. (a, b) GC-MS analysis of *Arabidopsis* roots in control condition (pink), metazaclor (Mz)-treated roots (green) and Mz-treated roots implemented with 24:0 (purple) (n = 4 biological replicates, the dots show the dispersion of data). (a) Quantification of non-α-hydroxylated fatty acids and (b) α-hydroxylated fatty acids. (c) Confocal micrographs of *Arabidopsis* root epidermal cells expressing the PI4P biosensor mCIT-3xPH^{FAPP1} upon 0, 50 Mz (Mz) treatment or 50 nM Mz treatment + 24:0 add-back. (d) Quantification of the fluorescence intensity at intracellular dots. While metazachlor treatment induces PI4P accumulation in intracellular dots, external application of 24:0 does not rescue this defect. The data are from the same result set as Figure 4. n = 75 cells from 25 roots for each condition. Statistics were done by two-sided Dwass-Steel-Critchlow-Flinger multiple comparison test with Monte Carlo method (10000 iterations). *** *P*-value <0.0001. Each element of the boxplot indicates the following value: center line, median; box limits, the first and third quartiles; whiskers, 1.5x interquartile range; points above or below the whiskers, outliers.

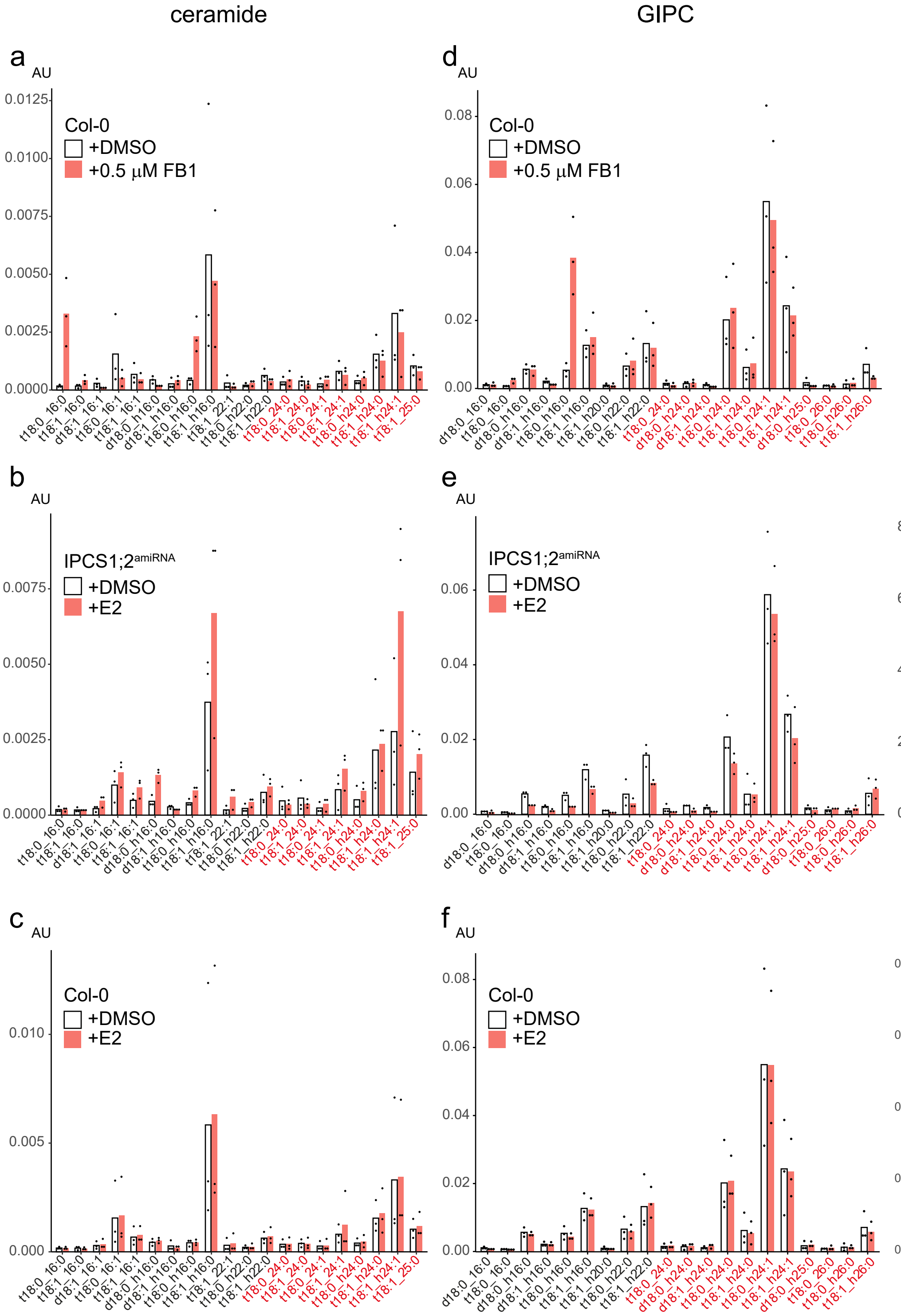

Supplementary Fig. 5. Additional information of figure 6b, c, d. This is the detailed LC-MS/MS sphingolipidomics of *IPCS1;2^{amiRNA}* line and FB1-treated *Arabidopsis* roots (n = 3 biological replicates, the dots show the dispersion of data, AU: Arbitrary Unit (area of peak compound/area of internal standard per mg fresh weight)). (a-c) Quantification of ceramide species. (a) FB1 treatment decreases VLCFA-ceramides, more particularly the t18:1 h24:0 and t18:1 h24:1 species, and increases 16-ceramides, especially t18:0 16:0 and t18:0 h16:0 species. (b) In the *IPCS1;2^{amiRNA}* line induced with β-estradiol (E2), all ceramide species increase and more particularly the t18:1 h16:0 and t18:1 h24:1 species. (c) In wild-type, the β-estradiol (E2) treatment does not modify the ceramide species profile. (d-f) Quantification of GIPC species. (d) FB1 treatment increases t18:0 h16:0 specie in the GIPC pool. (e) In the *IPCS1;2^{amiRNA}* line induced with β-estradiol (E2), all GIPC species decrease while (f) in wild-type, the β-estradiol (E2) treatment does not modify the GIPC species profile. (g-i) Quantification of GluCer species. (g) FB1 treatment increases t18:1 h16:0 specie in the GluCer pool. (h) In the *IPCS1;2^{amiRNA}* line induced with β-estradiol (E2), all GluCer species decrease while (i) in wild-type, the β-estradiol (E2) treatment does not modify the GluCer species profile.

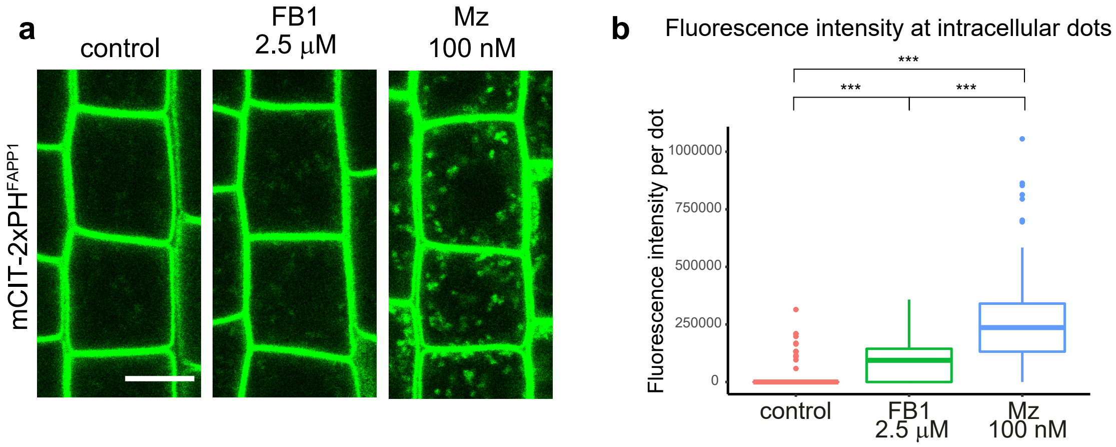

Supplementary Fig. 6. Fumonisin B1 treatment weakly induces PI4P accumulation at intracellular compartments as compared to metazachlor treatment. (a) Confocal micrographs of root epidermal cells expressing the PI4P sensor mCIT-2xPH^{FAPP1} in wild-type seedlings in control condition or upon FB1 or metazachlor (Mz) treatment. (b) Quantification of the fluorescence intensity at intracellular dots. FB1 induces significant accumulation of PI4P at intracellular compartments but this effect is weak as compared to Mz treatment. n = 60 cells from 20 roots for each condition. Statistics were done by two-sided Dwass-Steel-Critchlow-Flinger multiple comparison test with Monte Carlo method (10000 iterations). *** *P*-value <0.0001. Each element of the boxplot indicates the following value: center line, median; box limits, the first and third quartiles; whiskers, 1.5x interquartile range; points above or below the whiskers, outliers. Scale bar, 10 µm.

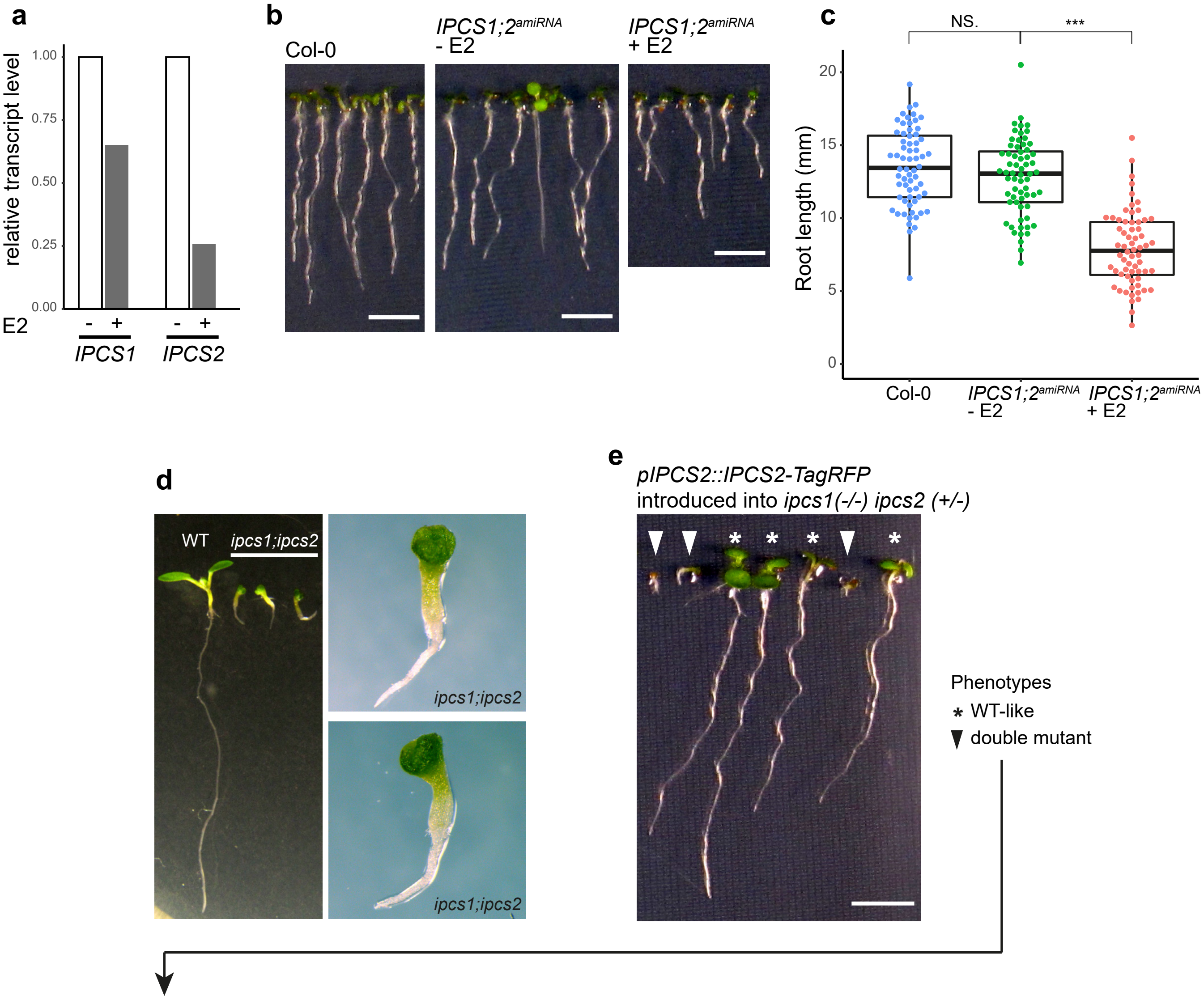

f T2 plants with *pIPCS2::IPCS2-TagRFP* introduced into *ipcs1(-/-) ipcs2 (+/-)* mutant line

Phenotype	Number of plants	Experimental segregation	Expected Mendelian segregation	
			non-complement	complement
* WT-like	244	94.6 %	75 %	93.75 %
▼ double mutant	14	5.4 %	25 %	6.25 %

Supplementary Fig. 7. Characterization of the *IPCS1;2^{amiRNA}* line and the pIPCS2::IPCS2-tagRFP construct. (a) RT-Q-PCR quantification of *IPCS1* and *IPCS2* mRNA level of expression in the *IPCS1;2^{amiRNA}* line in control condition or induced with β-estradiol (E2). Both *IPCS1* and IPCS2 level of expression is reduced in induced *IPCS1;2^{amiRNA}* line. n = 3 technical replicates. (b) Root growth phenotype of wild-type (Col-0), *IPCS1;2^{amiRNA}* line in control condition (- E2) or induced with β-estradiol (+ E2). (c) Quantification of the root length in (b). The root length of *IPCS1;2^{amiRNA}* line induced with β-estradiol (E2) is shorter as compared to *IPCS1;2^{amiRNA}* line in control condition or in wild-type. n = 64 roots for each condition. Statistics were done by two-sided Dwass-Steel-Critchlow-Flinger multiple comparison test with Monte Carlo method (10000 iterations). *** *P*-value <0.0001. Each element of the boxplot indicates the following value: center line, median; box limits, the first and third quartiles; whiskers, 1.5x interquartile range; points above or below the whiskers, outliers. (d) Seedling phenotype of the *ipcs1;ipcs2* constitutive double mutant. As compared to the wild-type (left), the *ipcs1;ipcs2* constitutive double mutant display very short roots, short hypocotyls and fused cotyledons. (e, f) The *pIPCS2::IPCS2-tagRFP* construct rescues the *ipcs1;ipcs2* constitutive double mutant phenotype. (e) Arrowheads indicates the *ipcs1;ipcs2* constitutive double mutants, the other seedlings are WT-like phenotype plants [ipcs1(-/-);IPCS2(+/+), ipcs1(-/-);IPCS2(+/-), or *ipcs1;ipcs2* constitutive double mutant showing the complementation of the phenotype by *pIPCS2::IPCS2-tagRFP* construct]. (f) Table of the segregation ratio of the phenotypes of the *Arabidopsis* line shown in (e). Scale bars, 5 mm.

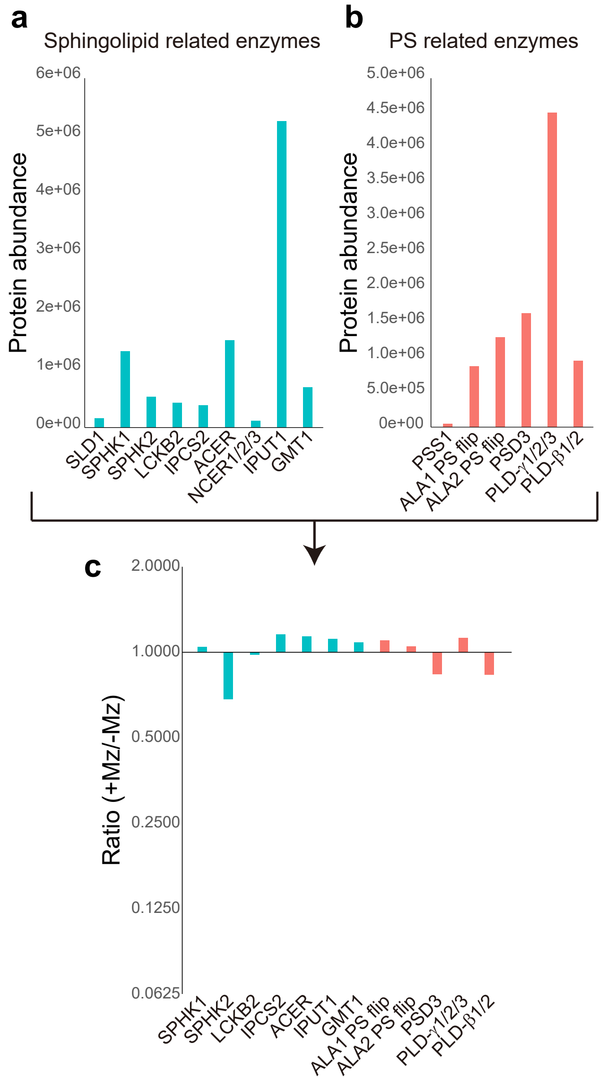

Supplementary Fig. 8. Immuno-purified SVs/TGN fraction contains a set of SL- and PS-related enzymes not targeted by acyl-chain length of SLs. (a, b) Proteomics results of sphingolipid or PS related enzymes. (a) Protein abundance of sphingolipid related enzymes. (b) Protein abundance of PS related enzymes. (c) The abundance ratio of the proteins shown in (a) and (b) between control and metazachlor (Mz)- treated sample. The same threshold as Figure 8 was applied. n = 4 biological replicates.

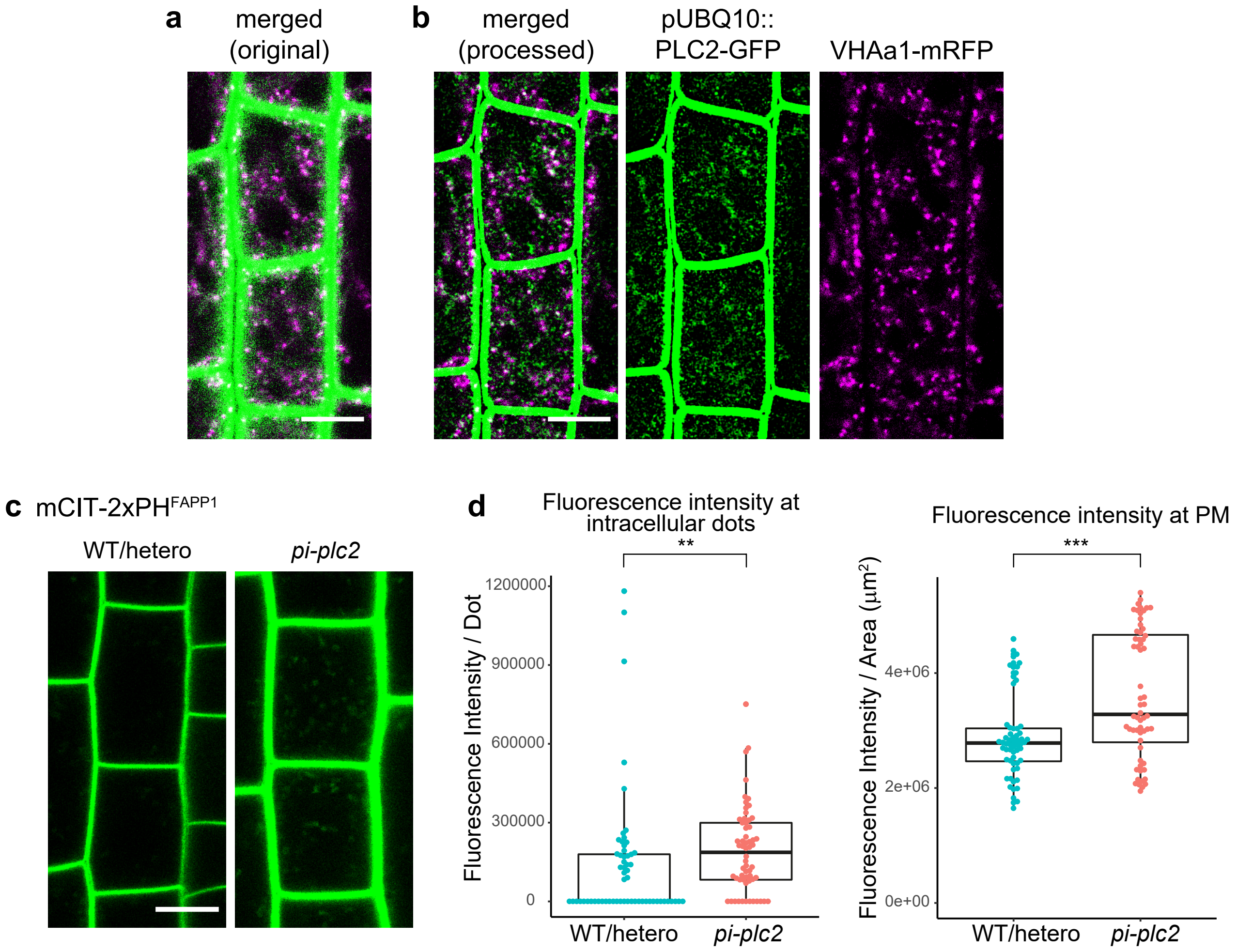

Supplementary Fig. 9. PI-PLC2 localizes at PM and SVs/TGN and is involved in PI4P accumulation at these compartments. (a) Original picture of pUBQ10::PLC2-GFP (green) co-localization with the SVs/TGN marker VHA-a1-mRFP. (b) Due to the low signal intensity of PLC2-GFP at intracellular compartments, the picture was processed with "subtract background" and "Gaussian filter" functions of Fiji software. PLC2-GFP structures partially co-localizes with VHA-a1-mRFP, the quantification of co-localization is displayed in Fig. 10. (c) Confocal micrographs of root epidermal cells expressing the PI4P sensor mCIT-2xPH^{FAPP1} in wild-type/heterozygotes or homozygotes *pi-plc2* mutant background. (d) Quantification of the fluorescence intensity at intracellular dots and. PI4P increases both at TGN and PM in *pi-plc2* mutant background. n = 60 cells from 20 roots. Statistics were done by two-sided Wilcoxson's rank-sum test. Each element of the boxplot indicates the following value: center line, median; box limits, the first and third quartiles; whiskers, 1.5x interquartile range; points above or below the whiskers, outliers. Scale bars, 10 µm.
