# Supplementary material for: Sphingolipids mediate polar sorting of PIN2 through phosphoinositide consumption at the trans-Golgi network
Source: Nat Commun. 2021 Jul 13;12:4267. doi: 10.1038/s41467-021-24548-0 (PMC8277843; doi:10.1038/s41467-021-24548-0)
Supplement: Supplementary file 3 — Descriptions of Additional Supplementary Files [file 41467_2021_24548_MOESM3_ESM.pdf]

Descriptions of Additional Supplementary Files

Supplementary Data 1
Description: P-values of all statistical tests used in this study.

Supplementary Data 2
Description: List of all primers used in this study.

Supplementary Data 3
Description: Full list of proteins found in the LC-MS/MS label-free quantitative proteomics of SVs/TGN immuno-purified compartments. This is the complete dataset for the proteomic analysis of SYP61-SVs/TGN compartments presented in Fig. 8 and Supplementary Fig. 8. Abundance values were calculated for each accession found (the number of peptides found is indicated) in each of the four biological repeats for both control condition and metazachlor (Mz) treatment. Average abundances for both control and Mz treatment and the ratio between Mz treatment and control were calculated resulting in a deprivation or enrichment value of a given protein in SYP61-SVs/TGN compartment upon Mz treatment. P-values of two-sided Wilcoxson's rank-sum test between the control and Mz treated samples are also presented.

Supplementary Data 4
Description: Short list of proteins displayed in this study and found in proteomics of SVs/TGN immuno-purified compartments. This is an extracted dataset of the proteins displayed in Fig. 8 and Supplementary Fig. 8 from Supplementary Data 3. Abundance values, average abundances for both control and metazachlor (Mz) treatment, the ratio between Mz treatment and control and Pvalues of two-sided Wilcoxson's rank-sum test are presented as in Supplementary Data 3.
