# Supplementary material for: Sphingolipids mediate polar sorting of PIN2 through phosphoinositide consumption at the trans-Golgi network
Source: Nat Commun. 2021 Jul 13;12:4267. doi: 10.1038/s41467-021-24548-0 (PMC8277843; doi:10.1038/s41467-021-24548-0)
Supplement: Supplementary file 8 — Reporting Summary [file 41467_2021_24548_MOESM8_ESM.pdf]

nature research

Corresponding author(s): Yohann Boutté

Last updated by author(s): Jun 21, 2021

Reporting Summary

Nature Research wishes to improve the reproducibility of the work that we publish. This form provides structure for consistency and transparency in reporting. For further information on Nature Research policies, see our Editorial Policies and the Editorial Policy Checklist.

Statistics

For all statistical analyses, confirm that the following items are present in the figure legend, table legend, main text, or Methods section.

n/a	Confirmed	
☐	☒	The exact sample size (n) for each experimental group/condition, given as a discrete number and unit of measurement
☐	☒	A statement on whether measurements were taken from distinct samples or whether the same sample was measured repeatedly
☐	☒	The statistical test(s) used AND whether they are one- or two-sided<br>*Only common tests should be described solely by name; describe more complex techniques in the Methods section.*
☒	☐	A description of all covariates tested
☒	☐	A description of any assumptions or corrections, such as tests of normality and adjustment for multiple comparisons
☐	☒	A full description of the statistical parameters including central tendency (e.g. means) or other basic estimates (e.g. regression coefficient) AND variation (e.g. standard deviation) or associated estimates of uncertainty (e.g. confidence intervals)
☐	☒	For null hypothesis testing, the test statistic (e.g. F, t, r) with confidence intervals, effect sizes, degrees of freedom and P value noted<br>*Give P values as exact values whenever suitable.*
☒	☐	For Bayesian analysis, information on the choice of priors and Markov chain Monte Carlo settings
☒	☐	For hierarchical and complex designs, identification of the appropriate level for tests and full reporting of outcomes
☒	☐	Estimates of effect sizes (e.g. Cohen's d, Pearson's r), indicating how they were calculated

Our web collection on statistics for biologists contains articles on many of the points above.

Software and code

Policy information about availability of computer code

Data collection	Araport protein database (version 11), NIST/EPA/NIH Mass spectral library (version NIST14)
Data analysis	ImageJ (version 1.53c), GEASE (version), MultiQuant (version 3.0, ABSciex), MassHunter qualitative analysis (version B.07.00, Agilent), Proteome discoverer (version 1.4, Thermo Fisher Scientific), percolar algorithm (MatrixScience), Progenesis QI for Proteomics (version 2.0, Nonlinear Dynamics).

For manuscripts utilizing custom algorithms or software that are central to the research but not yet described in published literature, software must be made available to editors and reviewers. We strongly encourage code deposition in a community repository (e.g. GitHub). See the Nature Research guidelines for submitting code & software for further information.

Data

Policy information about availability of data

All manuscripts must include a data availability statement. This statement should provide the following information, where applicable:

- Accession codes, unique identifiers, or web links for publicly available datasets
- A list of figures that have associated raw data
- A description of any restrictions on data availability

Data supporting the findings of this work are available within the paper and its Supplementary Information files. The mass spectrometry proteomics data have been deposited to the ProteomeXchange Consortium via the PRIDE partner repository with the dataset identifier PXD026252 [https://www.ebi.ac.uk/pride/archive/projects/PXD026252]. All other datasets and plant materials generated and analyzed during the current study are available from the corresponding author upon request. Source data are provided with this paper.

Field-specific reporting

Please select the one below that is the best fit for your research. If you are not sure, read the appropriate sections before making your selection.

☒ Life sciences ☐ Behavioural & social sciences ☐ Ecological, evolutionary & environmental sciences

For a reference copy of the document with all sections, see nature.com/documents/nr-reporting-summary-flat.pdf

Life sciences study design

All studies must disclose on these points even when the disclosure is negative.

Sample size	Proteomics and lipidomics experiments were performed with a number of replicates comprised between 3 and 5, this limited number of replicates is due to the extensive preparation time to produce these samples. For all the other experiments, we first estimated the necessary sample size, using the power.t.test function of R software, for two-sided t-test with the data of mCitrine-PH2xFAPP treated with 100 nM metazachlor compared to its control condition by setting the significance level at 0.01 and detection power at 0.8. Since the estimated minimum sample size was approximately 16, we decided to make the sample sizes more than 20. For the actual statistical test to show in the manuscript, we used non-parametric tests for all statistical analyses to get the highest stringency.
Data exclusions	No data were excluded from the analyses
Replication	All attempts of replication were successfull.
Randomization	Randomization was not practical due to the nature of the experimental setup.
Blinding	Blinding was not practical due to the nature of the experimental setup.

Reporting for specific materials, systems and methods

We require information from authors about some types of materials, experimental systems and methods used in many studies. Here, indicate whether each material, system or method listed is relevant to your study. If you are not sure if a list item applies to your research, read the appropriate section before selecting a response.

Materials & experimental systems

n/a	Involved in the study	
☐	☒	Antibodies
☒	☐	Eukaryotic cell lines
☒	☐	Palaeontology and archaeology
☒	☐	Animals and other organisms
☒	☐	Human research participants
☒	☐	Clinical data
☒	☐	Dual use research of concern

Methods

n/a	Involved in the study	
☒	☐	ChIP-seq
☒	☐	Flow cytometry
☒	☐	MRI-based neuroimaging

Antibodies

Antibodies used	Rabbit anti-ECHIDNA (not commercially available, this was a gift from the lab of Rishi Bhalerao UPSC, Umea, Sweden; published in Gendre et al., 2011, PNAS, 10:8048-53), rabbit anti-CHC (clathrin heavy-chain 1,2 from Agrisera, AS10 690), rabbit anti-MEMBRIN11 (not commercially available, this antibody was generated in our lab; published in Marais et al., 2015, J Ex Bot, 66:6665-78), rabbit anti-GFP (ThermoFisher Scientific, A-11122), mouse anti-GFP (Merck, 11814460001 Roche), TRITC-coupled donkey anti-rabbit IgG (Jackson Immunoresearch, 711-025-152), AlexaFluor 647 (A647)-coupled donkey anti-rabbit IgG (Jackson Immunoresearch, 711-605-152), goat anti-mouse IgG-HRP conjugate (Bio-Rad, 1721011), anti-rabbit IgG-HRP conjugate (Bio-Rad, 1706515).
Validation	Rabbit anti-ECHIDNA: this antibody was validated in echidna mutant background (Gendre et al., 2011, PNAS, 10:8048-53).<br>Rabbit anti-CHC reactivity: Arabidopis thaliana, Chlamydomonas reinhardtii, Nicotiana tabacum. KLH-conjugated peptide derived from available plant clathrin heavy chain sequences including Arabidopsis thaliana clathrin heavy chain 1 UniProt: Q0WNJ6, TAIR:At3g11130, clathrin heavy chain 2 UniProt: Q0WLB5,TAIR:At3g08530.<br>Rabbit anti-MEMBRIN11: this antibody was validated by immuno-precipitation and mass spectrometry (Marais et al., 2015, J Ex Bot, 66:6665-78).<br>Rabbit anti-GFP reactivity: tag. This antibody was verified by relative expression to ensure that the antibody binds to the antigen stated. Unconjugated polyclonal IgG.<br>Mouse anti-GFP reactivity: tag. This antibody is a monoclonal IgG1k from clones 13.1 and 7.1. Both Anti-GFP mouse monoclonal antibodies (Clones 7.1 and 13.1) are >95% pure as determined by SDS-PAGE and ion-exchange HPLC analyses.
